# Analyzing Thermal Degradation Effects on Devulcanized GTR-Based NR/SBR/NBR Rubber Compounds Reinforced with SiO_2_ Particles

**DOI:** 10.3390/polym16233270

**Published:** 2024-11-24

**Authors:** Xavier Colom, Laia Farrés, Ramon Mujal, Shifeng Wang, Javier Cañavate

**Affiliations:** 1 Department of Chemical Engineering, Universitat Politècnica de Catalunya Barcelona Tech, C/Colom, 1, 08222 Terrassa, Barcelona, Spain; laia.farres@estudiantat.upc.edu (L.F.); francisco.javier.canavate@upc.edu (J.C.); 2 Department of Electrical Engineering, Universitat Politècnica de Catalunya Barcelona Tech, C/Colom, 1, 08222 Terrassa, Barcelona, Spain; mujal@ee.upc.edu; 3 School of Chemistry and Chemical Engineering, Shanghai Jiao Tong University, Shanghai 200240, China; shfwang@sjtu.edu.cn

**Keywords:** ground tire rubber (GTR), natural rubber (NR), styrene–butadiene rubber (SBR), nitrile butadiene rubber (NBR), SiO_2_, TESPT, devulcanization process

## Abstract

The large number of tires produced annually demands new recycling methods. A key challenge associated with recycling elastomers is their crosslinking structure that prevents fusion. It is possible to reverse crosslinking through a process called devulcanization. Devulcanized elastomers can be blended with fresh rubber and revulcanized for reuse. This paper examines samples made from natural rubber (NR), styrene–butadiene rubber (SBR), and nitrile butadiene rubber (NBR), blended with varying proportions of devulcanized ground tire rubber (dGTR) and newly revulcanized rubber. SiO_2_, commonly present in tire formulations, is also added. Samples of these materials, with 0, 10, 20, and 40 phr of dGTR are subjected to accelerated degradation for 0, 30, 60, 120, and 240 h. The effects of this treatment, the influence of SiO_2_, and the presence of a silane-based devulcanization agent (TESPT) that promotes the interaction between the rubber and silica, are analyzed at the microstructural level (FTIR, TGA, SEM) and through mechanical properties testing. The microstructural results of the spectroscopy and thermal analysis show that interactions between dGTR, silica, and silane compounds form aggregates that impact the material properties and degradation of the tires. Mechanically, when the sample contained up to 20 phr of dGTR, the compound presented a more brittle behavior, due to the crosslinking induced by the degradation.

## 1. Introduction

Green rubber compounds present a promising solution to recover a significant portion of the one billion end-of-life tires generated globally each year [1]. In Europe alone, around 4.2 million tons of used tires were generated in 2023, according to the European Tyre and Rubber Manufacturers’ Association (ETRMA) [2]. However, only 600,000 tons are reused or retreaded, leaving 3.6 million tons classified as end-of-life tires. This substantial accumulation of used tires highlights the urgent need for valorization strategies to: (i) reduce landfill waste, (ii) prevent fires, (iii) conserve natural resources, (iv) lower water and energy consumption, (v) minimize the carbon footprint, and (vi) promote a sustainable economy, while protecting biodiversity.

As residua, used tires are especially difficult to deal with because they are bulky, non-biodegradable, and occupy significant space in landfill sites, where they generate leachates and release harmful chemicals, posing serious environmental risks. Additionally, they can become breeding grounds for disease-carrying mosquitoes and are highly flammable, increasing the risk of fires that emit toxic gases and present a significant threat to public health.

Furthermore, tire recycling should be a more energy-efficient process than manufacturing new tires, leading to significant reductions in fossil fuel consumption and greenhouse gas emissions. Producing one ton of new tires requires 21.7 MWh of energy, whereas recycling this amount of tires would likely offer substantial energy savings in comparison to this data, helping to reduce the industry’s carbon footprint. According to the United States Tire Manufacturers Association (USTMA) [3], in the event of increased tire recycling, CO_2_ emissions would drop from 63.64 kg to 30.58 kg per ton of tires, representing a 52% reduction, while the demand for virgin raw materials would decrease by 45%, conserving resources and reducing the reliance on non-renewable sources.

Among tire recycling methods, devulcanization has gained particular attention because of its conception based on breaking the crosslinked structure of elastomers, by cleaving the sulfur bonds formed during vulcanization [4,5]. Devulcanization enables the reprocessing of rubber, allowing it to be blended with new materials, such as styrene–butadiene rubber (SBR), natural rubber (NR), and nitril rubber (NBR), which is common in tire production. The blend can be posteriorly revulcanized again. This process offers a way to reintegrate waste rubber into new tire manufacturing, while maintaining similar material properties. Ground tire rubber (GTR), a powder produced from recycled car and truck tires, is generally the starting material in the devulcanization process [6]. The subsequent product, devulcanized GTR (dGTR) is then blended and revulcanized with the other components in order to produce tire rubber [7].

The properties of blends incorporating dGTR and the degradation that tires undergo during their service life are still underexplored. This degradation can cause significant changes in the rubber’s properties and these alterations may affect the performance of new tires made with devulcanized GTR. Understanding the impact of this degradation is essential to ensure the viability of using devulcanized rubber in tire manufacturing [8].

The efforts to reduce CO_2_ emissions and advance towards a more sustainable economy are leading to the adoption of electric vehicles (EVs). EVs produce no direct emissions, which lowers their carbon footprint. However, due to the increased weight of electric cars, tire formulations have had to be adapted. A significant proportion of silica (up to 30 phr) has been incorporated into tires for electric vehicles. Studies show that the addition of silica (SiO_2_), high-defect multiwalled carbon nanotubes (HD-CNT), and carbon black (CB), in the formulation of these elastomeric materials, enhances their mechanical properties, thermal conductivity, and volume resistivity [9]. Silica, in particular, offers benefits, such as improved fuel efficiency and better grip, especially in wet conditions [10,11].

The challenge with using an increased proportion of silica nanoparticles is their poor dispersion within the elastomeric matrix, primarily due to the differences in polarity between the two materials that can result in the formation of agglomerates, reducing the effectiveness of silica reinforcement. To address this issue, silanes are commonly used as coupling agents. These compounds have functional groups that bond with both the elastomer matrix and the silica particles, improving their dispersibility and enhancing the overall performance [12,13,14].

In this study, the POLQUITEX research group, with proven experience in polymer aging processes [15], aims to investigate vulcanized blends that mimic the composition of a tire that incorporates 0, 10, 20, and 40 phr of devulcanized ground tire rubber (dGTR), styrene–butadiene rubber (SBR), natural rubber (NR), and nitril rubber (NBR). These blends are also degraded for 0, 30, 60, 120, and 240 h. The effect of different dGTR content and compound compositions, the impact of the addition of SiO_2_, the changes in the tensile behavior, and the influence of degradation over time, will be analyzed.

## 2. Materials and Methods

### 2.1. Materials

NR SVR CV (Vietnam natural rubber), with ash content at a max. of 0.4% wt, volatile matter at 0.8% wt, with a Plasticity Retention Index (PRI) of 60, and a density of 0.92 g/cm^3^; SBR Pliogum 1027K1, with 24 wt.% of bonded styrene (MV ML1+4, 100 °C; 52); and NBR Ker N-29, with 29 wt.% of bonded acrylonitrile (MV ML1+4, 100 °C; 50); were supplied by VIGAR HEXPOL Compounding SLU, Rubí (Spain).

Ground tire rubber (GTR) is obtained from the shredding of the tires of vehicles in special processing plants. In this case, the GTR is a mix of passenger and truck tires, with particle sizes of up to 0.7 mm and an average particle size of 320 microns, which were received by Gestión Medioambiental de Neumáticos S. L. (GMN) in Maials (Spain). The composition of the rubber, according to our thermogravimetric analysis, is natural rubber 29%; synthetic rubber 31%; carbon black 29.5%; SiO_2_ 7%; other additives (e.g., curing system, processing aids) 3.5%; which is very close to the composition of the rubber obtained by Li et al. [16]. Details on the particle size distribution have been published previously by Colom et al. [17].

The additives and curing agents used for curing, namely vulcanization accelerators (TBBS N-cyclohexil-2-benzotiazolsulfenamide, TMTD—tetramethylthiuram disulfide), carbon black N550, stearic acid, zinc oxide, sulfur with technical purity, and SiO_2_ (85% particle size below 150 microns), were supplied by VIGAR Rubí (Spain).

Bis (3-triethoxysilyl propyl) tetrasulfide (TESPT) activated the devulcanization. It was also supplied by VIGAR Rubí (Spain).

### 2.2. Devulcanization of GTR

The devulcanization process for GTR using combined thermo-mechanical/chemical procedures was carried out by modifying a procedure already tested in similar cases and published by our research group in a previous paper [18].

The thermo-mechanical/chemical treatment is carried out using GTR, with the addition of 2 parts per hundred rubber (phr) of TESPT, which acts as a devulcanizing agent [19]. This blend is passed through the machine 10 times at 50 °C and 80 rpm. The sample, after this treatment, presents as putty-like when touched, an aspect which is often observed in devulcanized rubber.

### 2.3. Preparation of Rubber Compounds

The rubber compounds were prepared using the formulation presented in Table 1. The first step in the mixing process consists of the preliminary plasticization of the fresh elastomeric compounds, NR, SBR, and NBR, for 5 min. After that, the mixing stage takes place, involving devulcanized GTR, 30 phr of carbon black, and 30 phr of SiO_2_, for 5 min. Finally, the sulfur curing system (zinc oxide 5.0 phr; stearic acid 3.0 phr; TBBS 1.0 phr; TMTD 0.25 phr; sulfur 2.0 phr) is added to the blend and mixed for 2 min, allowing the revulcanization process to take place.

The rationale behind the studied formulations is to maintain the overall composition of a typical tire, while substituting only the elastomers with those found in devulcanized ground tire rubber (dGTR). The baseline tire composition is defined in the initial formulation. For example, when 10 phr of dGTR is added, it introduces not only elastomers, in the same initial proportion, but also, according to the composition of a typical tire, 0.7 phr of SiO_2_ and 3 phr of carbon black (CB). To preserve the original proportions, we adjust these components accordingly, reducing SiO_2_ by 0.7 phr and CB by 3 phr for every 10 phr of dGTR added. The remaining components, associated with the curing system, are unaffected by the addition of dGTR and are incorporated in the same proportions as in the original blend.

The rubber ingredients were mixed in a Plasticorder internal mixer (Brabender GmbH & Co. KG, Duisburg, Germany) at 80 °C and 80 rpm. The order of appearance of the components presented in Table 1 (left to right) also reflects the order of mixing. The devulcanized GTR content in the samples was 0, 10, 20, and 40 parts per hundred rubber (phr).

Two batches of the mix, cited above, are necessary to create a suitable sample plate of the polymer. These two batches are recombined in a two-roll mill Collin Teach-line press. The resulting material is subsequently molded in a Collin P 200E hot plate press at 160 °C and 200 bar for 12 min [20]. These conditions correspond to the optimum curing time (t90), determined by the vulcanization characteristics of vulcanizable rubber composites, according to ASTM D2084 [21], with a Monsanto Oscillating Disc Rheometer R 100 (Akron, OH, USA), at 162 ± 1 °C. After this time, the mold is removed carefully from the press, using Kevlar gloves, and quickly taken away to cool. Finally, the mold is separated from the part, which is now ready to shape into the test samples with a J.BOT Instruments S.A. (Barcelona, Spain) test cutter, designed to obtain dumbbell-shaped test samples, according to the specifications in ASTM D-412 [22].

### 2.4. Thermo-Oxidation Process

The aging treatment was performed in an air-forced oven at 80 °C (Selecta, Barcelona, Spain). The samples were exposed to the conditions for different time periods (0, 30, 60, 120, and 240 h), according to ISO 4577 [23].

### 2.5. Measurements

The tensile properties of the vulcanized rubber dGTR/NR/SBR/NBR composites were evaluated according to the ISO 37 [24]. Testing was conducted using a high-precision Instron 3366 testing machine from Norwood, MA (USA), with a pneumatic cell load capacity of 2 kN. The tensile tests were executed at a constant cross-head speed of 500 mm/min, in a controlled environment, with a relative humidity (RH) of 50 ± 5% and a temperature of 23 ± 2 °C. Five specimens per test were used.

The chemical structure of the samples was determined using FTIR–ATR analysis, performed by means of a Spectrum Two spectrometer from Perkin Elmer (Waltham, MA, USA). The device had a diamond ATR crystal attachment. The spectra were registered at a resolution of 2 cm^−1^ and 40 scans took place in the range of 3500–500 cm^−1^, according to which the compound signals related to different deformation bands can be observed.

The TGA was performed using Perkin Elmer TGA 8000 apparatus (USA). A sample of the composites, weighing approximately 10 mg, was placed in a corundum dish. The measurement was conducted in the temperature range 30–800 °C and under atmospheric oxidation with air (30 mL/min), at a heating rate of 20 °C/min.

The fracture surface of the samples, as a result of mechanical testing, was examined with a JEOL JSM 6480 scanning electron microscope from Akishima, Tokyo, Japan. The dry samples were coated with a thin layer of gold before observation under the microscope, to increase the sample conductivity.

## 3. Results

### 3.1. Mechanical Properties

**Figure 1:**
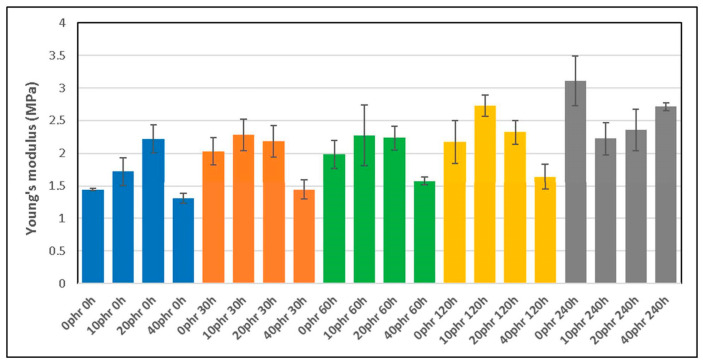
Young’s modulus (YM) of different compounds as a function of dGTR content and thermo-oxidative exposure time.

As can be seen in Figure 1, Young’s modulus (YM) follows an increasing trend as a function of the exposure time to thermo-oxidative aging. Likewise, it can also be observed that for the same exposure time, the trends change as a function of the dGTR content of the samples. Some of the samples with an exposure time of 120 h, have YM values that show increases of 140%. This is due to two phenomena, namely the formation of crosslinks in the thermo-oxidated elastomeric chains and the generation of hard and brittle layers around the particles, as has been reported in previous studies [25,26,27].

The brittleness of these samples is due to different processes: (a) the devulcanization of the GTR particles in the presence of silane-based TESPT induces a significant affinity of the devulcanized GTR to the SiO_2_ micro-fillers, forming an aggregate of TESPT–SiO_2_ around the dGTR particles; (b) this aggregate prevents, at least partially, the exudation of carbon black particles to the surface, caused by the increase in temperature to 80 °C, and they are placed between the interstitial spaces of the TESPT–SiO_2_, forming a hard and brittle layer; and (c) when the dGTR content is lower than 20 phr, YM grows significantly, above this the samples present brittle behavior that decreases the mechanical properties. With the increased exposure time, the amount of CB diffused increases and, consequently, the thickness of the layer around the dGTR also increases.

**Figure 2:**
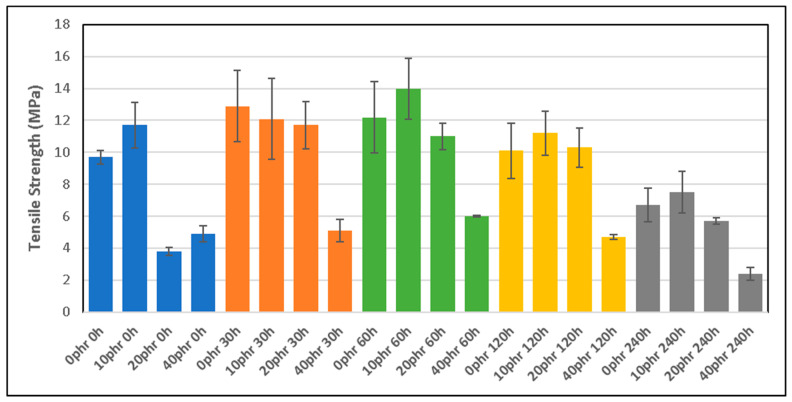
Tensile strength (TS) of different compounds as a function of dGTR content and thermo-oxidative exposure time.

Figure 2 presents the tensile strength (TS) values as a function of the thermo-oxidative aging exposure time and dGTR content (phr). The results show an increase in the TS up to 60 h of exposure, with dGTR content of 20 phr. It is interesting to see how, in all cases, a dGTR content of 40 phr causes a significant decrease in the TS. This is due to the presence of large agglomerates of dGTR wrapped in TESPT–SiO_2_ and CB, which generate a stress nucleus, where the samples easily break, and are produced when the content of dGTR increases. It can also be concluded that aging for 60 h does not alter the TS. Comparing these samples (dGTR/NR/SBR/NBR/SiO_2_) with the samples dGTR/NBR/SiO_2_, it can be seen that they have similar tensile properties [26]. It should also be considered that when the size of the dGTR particles is large, the affinity between the elastomeric matrix and the dGTR is low and causes premature breakage of the samples [27].

**Figure 3:**
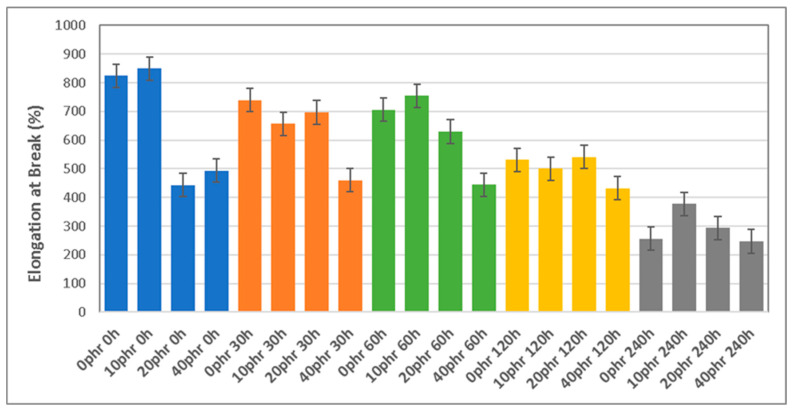
Elongation at break (EB) of different compounds as a function of dGTR content and thermo-oxidative exposure time.

The elongation at break (EB) as a function of the aging time (Figure 3) follows a fairly defined decreasing trend. Higher EB values are observed for the samples with 0 and 10 phr dGTR, up to an exposure time of 60 h. Between 120 and 240 h, the decrease in the elongation at break is significant. In samples with 20 and 40 phr dGTR, a marked decrease is also observed, mainly for the non-aged samples. The behavior at the EB is also explained by the same phenomena discussed previously.

**Figure 4:**
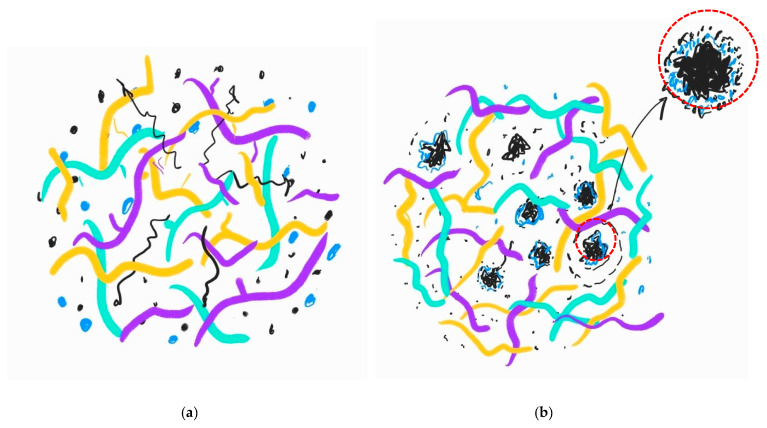
Proposal squema showing the phenomenon of CB and SiO_2_ migration from the core to the surface in: (**a**) the NR/SBR/NBR composite without dGTR and (**b**) composites with dGTR. Blue strands correspond to SBR, yellow strands to NR, lilac strands to NBR, black dots to CB, and blue dots to SIO_2_. The enlarged red circle shows the SiO_2_ evolved dGTR particle that prevents CB exudation, generating a brittle layer of CB.

The diagram in Figure 4 shows the different phases of the phenomenon that takes place in the compounds without and the compounds containing dGTR particles, neat elastomers (NR, SBR, and NBR), 30 phr of CB, and 30 phr of SiO_2_. It can be observed how the dGTR, due to the presence of TESPT, retains SiO_2_ micro-fillers in a wrapped manner, which at the same time prevents the exudation of CB towards the surface, forming SiO_2_–CB agglomerates, which link multiple particles around the dGTR. These larger aggregates significantly weaken the structure of the sample [28].

### 3.2. FTIR Spectroscopy Characterization

**Figure 5:**
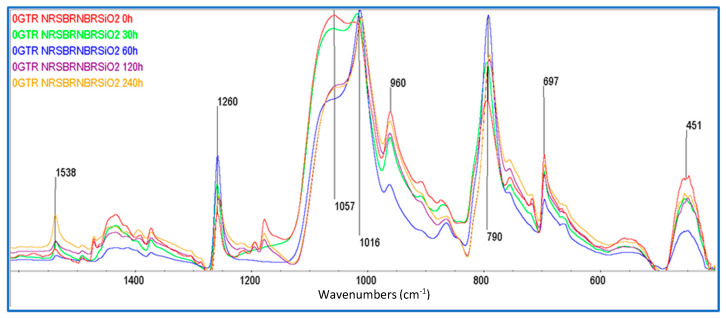
FTIR spectra of NR/SBR/NBR/SiO_2_ compounds as a function of different thermo-oxidation exposure times (0, 30, 60, 120, and 240 h). Y axis in arbitrary units.

As can be observed in the spectra in Figure 5, the most interesting bands to be analyzed are those corresponding to SiO_2_ at 1057 cm^−1^ and carbon black (CB) at 1016 cm^−1^. The samples without dGTR present an intense band corresponding to SiO_2_ in the unaged samples. The band assigned to CB at 1016 cm^−1^ is intense and is not altered by the degradation time. As mentioned in regard to the measurements, the spectra were recorded by ATR spectroscopy and, as such, are representative of the surface of the samples. The difference in the variations in the intensity of those two bands is then related to the diffusion phenomena. The band assigned to CB remains at a high and constant intensity because the nanometric size of this filler gives it a high capacity to diffuse through elastomeric structures in order to reach the surface; the amount of CB observed in the spectra is not altered by the degradation time. Instead, the SiO_2_ crystals, originally dispersed within the elastomeric matrix, diffuse towards the surface with a longer degradation time. After 60 h, the elastomeric structures of NR (which are less stable than those of SBR and NBR) [29] begin to degrade, generating a greater number of branches, oxidation groups, and crosslinks. These structural changes, together with the abovementioned effect of TESPT attaching, prevent greater diffusion of SiO_2_ towards the surface, which is observed with a lower band intensity.

The band at 1538 cm^−1^, assigned to ZnSt_2_, increases its intensity with long aging times. The diffusion of ZnSt_2_ into similar elastomeric samples has been studied previously [30,31]. The particles of ZnSt_2_, at a degradation temperature of 80 °C, can diffuse towards the surface and the degradation of the material improves the particles’ ability to move through the structures. The other bands, 960, 790, 697, and 451 cm^−1^, are assigned to -CH=CH-, trans-polybutadiene, benzenic rings, and S-S, respectively. The intensity of each band depends on the number of groups that appear at the surface [32,33,34,35].

**Figure 6:**
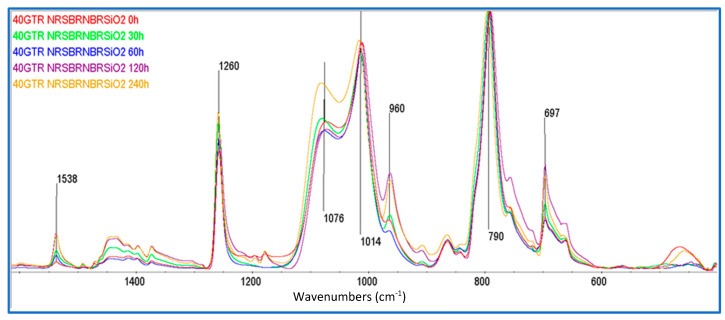
FTIR spectra of dGTR/NR/SBR/NBR/SiO_2_ compounds filled with 40 phr of dGTR as a function of different thermo-oxidation exposure times (0, 30, 60, 120, and 240 h). Y axis in arbitrary units.

There is an interesting effect in regard to the SiO_2_ band at 1057 cm^−1^ that further corroborates the previous discussion and is present in Figure 6. When dGTR is added to the rubber compound (RC) formulation, the band at 1057 cm^−1^ shifts to 1076 cm^−1^, and its intensity decreases for all aging periods (Figure 6). This is a consequence of the adhesion or wrapping effect on the SiO_2_ particles caused by the affinity produced by the coupling agent TESPT, which causes the adhesion of the SiO_2_ particles to the surface of the dGTR. Furthermore, according to Ghorai et al. [19], devulcanized GTR naturally has much more of an affinity for SiO_2_ particles than for other particles in the sample, such as CB or ZnSt_2_. Likewise, as represented in the squama in Figure 4, both particles can be retained by the SiO_2_ aggregates that form around the dGTR. The spectral intensity of these bands (CB and ZnSt_2_) decrease in the RC with dGTR, in comparison with the RC without dGTR.

### 3.3. Thermogravimetric Analysis

**Figure 7:**
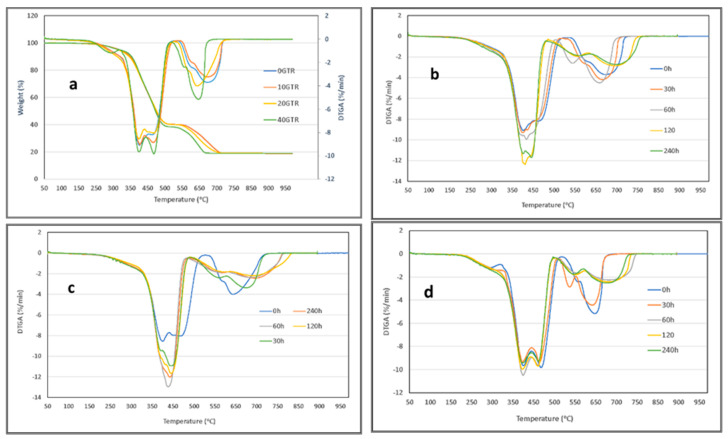
(**a**) TGA and DTGA of dGTR/NR/SBR/NBR/SiO_2_ compounds filled with 0, 10, 20, and 40 phr of dGTR; (**b**–**d**) DTGA of dGTR/NR/SBR/NBR/SiO_2_ compounds filled with 0, 20, and 40 phr of dGTR as a function of different thermo-oxidation exposure times (0, 30, 60, 120, and 240 h).

Figure 7a shows the TGA thermograms of the dGTR/NR/SBR/NBR/SiO_2_ compounds, illustrating various dGTR and silica loadings. The weight loss percentages of the samples with different dGTR content are shown, revealing a consistent residue value of 20%, attributed to SiO_2_. The DTGA results indicate a heterogeneous elastomeric composition (NR/SBR/NBR), where each component exhibits distinct thermal decomposition peaks at different temperatures. In samples with low dGTR content (0% and 10%), three peaks are identifiable: the first corresponds to NR (370 °C), the second to SBR (445 °C), and the third to NBR (455 °C). As the dGTR content increases, the thermal decomposition temperature (TdT) of SBR and NBR join into a single domain above 450 °C, while NR remains clearly above 400 °C. It is also worth noting that the TdT of different components increases with silica [36].

NR and NBR are incompatible, resulting in the presence of distinct domains for both polymers in the compound. However, there is partial miscibility between NR and NBR (due to presence of SBR), leading to interactions and potential crosslinking of the polymer chains, which causes a shift in the peak temperatures. This is particularly evident for the NBR peak, which shifts from 485 °C (without NR) to approximately 460 °C, for a composition of 67% NR and 33% NBR [8].

As the dGTR amount increases, the significance of these peaks also rises. This is attributed to the dGTR, derived from recycled tires, which contains virgin elastomers that enhance the overall amount of elastomers in the sample. However, the degradation temperatures across the samples do not show substantial differences. The subsequent peak corresponds to carbon black (CB) [12], whose decomposition temperature varies among the different samples. Remarkably, samples with higher dGTR content (higher than 20% dGTR) decompose first, unlike those with lower dGTR amounts (0% and 10%). This trend is likely due to the increased formation of aggregates in the samples with higher dGTR, which reduces the degree of dispersion and significantly reduces the TdT range. The aggregates further associate into even larger structures, via van der Waals forces, to form agglomerates [11].

Figure 7b–d illustrates the DTGA curves of the 0, 20, and 40 phr dGTR thermo-oxidated compounds, revealing three distinct peaks. As previously mentioned, the first peak corresponds to NR, the second to the SBR–NBR domain, and the third to the TdT of carbon black (CB). The thermo-oxidation process significantly modifies these peaks, with the samples containing lower dGTR content (0 and 20 phr) being more affected than those with 40 phr (Figure 7d). This is because thermo-oxidation at 80 °C is more effective for samples with a higher raw elastomer content (NR, SBR, and NBR), compared to those with greater dGTR content.

Particularly, there are substantial differences in the broad peak of the TdT for CB among the samples without dGTR (Figure 7b), those with 20 phr dGTR (Figure 7c), and those with 40 phr dGTR (Figure 7d). In the samples without dGTR, the dispersion of CB nanoparticles is more uniform, and their mobility and infiltration within the silica matrix increase with the temperature and prolonged exposure time (up to 120 days). This enhanced dispersion contributes to a wider range of TdTs for these particles.

Alternatively, in samples with dGTR, the agglomerates formed around the dGTR limit the effectiveness of thermo-oxidation at shorter times (30–60 h), resulting in a narrower TdT range. The presence of dGTR (using TESP as a devulcanizing agent) facilitates the formation of SiO_2_ macroaggregates that encase the dGTR. In this scenario, more CB nanoparticles become entrapped within these macroaggregates, leading to a higher concentration of nanoparticles, which reduces the TdT range.

### 3.4. Scanning Electron Microscopy Analysis

**Figure 8:**
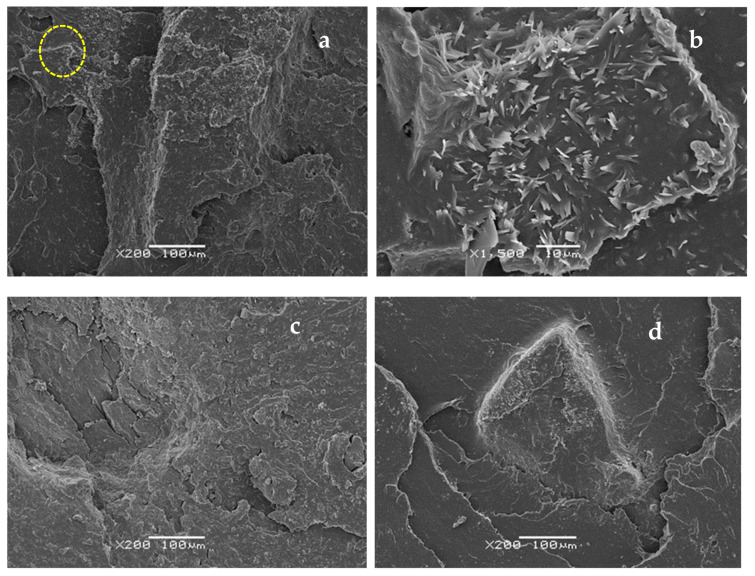
SEM photographs of fractured tensile compounds: (**a**) without dGTR, (**b**) magnification (×1500) of yellow circle, (**c**) with 40 phr dGTR at 0 h of thermo-oxidation, (**d**) with 40 phr dGTR at 120 h of exposure to the thermo-oxidation process.

The images in Figure 8 show the exudation process involving CB and SiO_2_ that has been explained previously. These images correspond to the fractured surface of the samples. In Figure 8a, which corresponds to the compounds without dGTR, it is observed that the SiO_2_ particles (more visible) have easily migrated to the surface of the sample. Figure 8b shows these particles in a maximized form and they correspond to SiO_2_ [37]. Figure 8c,d is related to the compounds with 40 phr dGTR submitted to 0 h and 120 h of thermo-oxidative degradation, in which it is observed that the SiO_2_ and CB particles are retained around the dGTR, forming agglomerates. In Figure 8d, this phenomenon is observed more intensely.

## 4. Conclusions

The results of the present study show that the effect of degradation of dGTR-based, sustainable, NR/SBR/NBR rubber compounds affects the mechanical properties of the materials. Young’s modulus increases with the thermo-aging exposure time, due to crosslink formation and the generation of hard, brittle layers around the dGTR particles. When the dGTR content exceeds 20 phr, the samples become more brittle. The tensile strength peaks at 60 h of exposure to thermo-aging in samples with 20 phr of dGTR content. Higher dGTR content (40 phr) leads to a decrease in the tensile strength. This is due to the effect of the silane-based devulcanizing agent that promotes interaction between the rubber and silica, generating large aggregates of dGTR wrapped in TESPT–SiO_2_ and CB, which create a stress nucleus and weak points. The elongation at break decreases significantly with increasing dGTR content and prolonged aging. The samples with 0–10 phr of dGTR showed higher elongation, while those with 20–40 phr exhibited a marked decrease, particularly after 120 h of exposure to the thermo-oxidation process.

Using FTIR spectroscopy, the different SiO_2_ and carbon black behaviors during degradation can be observed. SiO_2_ diffusion decreases over time, while the diffusion of CB remains constant. SiO_2_ particles form aggregates around the dGTR, due to the previously mentioned presence of a coupling agent (TESPT), which reduces diffusion, altering the mechanical properties of the materials. ZnSt_2_ particles, as corroborated by other studies, show increased diffusion toward the surface with longer aging times.

The thermogravimetric analysis showed the patterns of decomposition of the involved elastomers and carbon black. The samples with higher dGTR content decomposed faster due to the formation of aggregates, which limited the dispersion and reduced the thermal stability of the materials. The samples with lower dGTR content exhibited distinct peaks for NR, SBR, and NBR, while the samples with higher dGTR content had merged thermal decomposition peaks, which indicates compatibility among the elastomers composing the samples. Aggregates formed by SiO_2_-TESPT around the dGTR particles affect the dispersion of carbon black particles, which changes the thermal properties of the materials and results in a change to the thermal decomposition temperature.

## Figures and Tables

**Table 1:** Sample codes and composition of different compounds in phr (per hundred rubber).

Sample Code	NR	SBR	NBR	dGTR	SiO_2_	CB	S	ZnO	HSt	TBBS	TMTD
0GTRNRSBRNBR	50	35.0	15.0	0	30.0	30	2	5	3	1	0.25
10GTRNRSBRNBR	47	33.0	14.0	10	29.3	27	2	5	3	1	0.25
20GTRNRSBRNBR	44	31.0	13.0	20	28.6	24	2	5	3	1	0.25
40GTRNRSBRNBR	38	27.0	11.0	40	27.2	20	2	5	3	1	0.25

## Data Availability

The original contributions presented in the study are included in the article, further inquiries can be directed to the corresponding author.
